# Do Individuals With Obsessive-Compulsive Disorder and Obsessive-Compulsive Personality Disorder Share Similar Neural Mechanisms of Decision-Making Under Ambiguous Circumstances?

**DOI:** 10.3389/fnhum.2020.585086

**Published:** 2020-10-22

**Authors:** Yudan Luo, Lu Chen, Hongchen Li, Yi Dong, Xiaoqin Zhou, Linlin Qiu, Lei Zhang, Yaxiang Gao, Chunyan Zhu, Fengqiong Yu, Kai Wang

**Affiliations:** ^1^Department of Medical Psychology, School of Mental Health and Psychological Sciences, Anhui Medical University, Hefei, China; ^2^School of Mental Health and Psychological Sciences, Collaborative Innovation Centre of Neuropsychiatric Disorder and Mental Health, Hefei, China; ^3^School of Mental Health and Psychological Sciences, Anhui Province Key Laboratory of Cognition and Neuropsychiatric Disorders, Hefei, China; ^4^School of Civil Engineering, Wangjiang University of Technology, Ma’anshan, China; ^5^School of Mental Health and Psychological Sciences, Anhui Mental Health Center, Hefei, China; ^6^The Chaohu Affiliated Hospital of Anhui Medical University, Hefei, China; ^7^Department of Neurology, The First Affiliated Hospital of Anhui Medical University, Hefei, China

**Keywords:** obsessive-compulsive personality disorder (OCPD), decision-making, event-related potentials, obsessive-compulsive disorder (OCD), Iowa Gambling Task (IGT)

## Abstract

Impaired decision-making is well documented in obsessive-compulsive disorder (OCD) and a range of electrophysiological and functional neuroimaging measures have begun to reveal the pathological mechanisms that underlie the decision-making process. Obsessive-compulsive personality disorder (OCPD) has core symptoms that often overlap with OCD, but similarities between these disorders at the behavioral and neurological levels are often unclear, including whether OCPD exhibits similar decision-making deficits and shared neurological dysfunction. To address these issues, we examined 24 cases of OCD, 19 cases of OCPD, and 26 matched normal control (NC) subjects during the revised Iowa Gambling Task (IGT) using event-related potentials (ERPs). The net IGT scores were lower for OCD subjects than for OCPD or NC subjects, thus indicating that OCD subjects chose more disadvantageous options and were “short-sighted” with regards to information. The feedback-related negativity (FRN) waveform (lose-win) was larger in both OCD and OCPD subjects, which suggested that obstacles exist in the feedback process. Consequently, these subjects might share similar neural mechanisms under ambiguous decision-making circumstances. Furthermore, IGT net scores were significantly and negatively correlated with Hamilton Anxiety Rating Scale (HAMA) and Hamilton Depression Rating Scale (HAMD) scales. This implies that more severe obsessive-compulsive symptoms inspired more negative emotions that led to worse decision-making ability. Therefore, although similar neural mechanisms might exist, this led to different behaviors in which OCPD is associated with better behavioral performance compared to OCD patients.

## Introduction

Obsessive-compulsive disorder (OCD) and obsessive-compulsive personality disorder (OCPD) are two distinct disease entities according to the Fifth Edition of the Diagnostic and Statistical Manual of Mental Disorders (DSM-5; American Psychiatric Association, 2013). OCD is a form of “obsessive-compulsive and related disorders” and has a prevalence of approximately 1–3% (Ruscio et al., 2010). OCD is characterized by persistent, time-consuming, and distressing, obsessions and/or compulsions that disrupt numerous activities of daily living. Alternatively, OCPD is one of the first confirmed personality disorders with an approximate lifetime prevalence of 2–8% (Torgersen et al., 2001; Samuels et al., 2002; Grant et al., 2004), and is characterized by perfectionism, rigidity, preoccupation with details, and the need for mental and interpersonal control. Due to the overlap in clinical symptoms and difficulties in diagnosis, the distinction between OCD and OCPD is often unclear. Consequently, there is significant debate as to whether we should re-classify OCD and OCPD. Indeed, 47.3% of subjects with OCD meet the criteria for OCPD. Subjects with OCD and comorbid OCPD show an earlier onset, a greater severity, and a poorer treatment outcome (Starcevic et al., 2013). It is suggested that OCPD may be a subtype of OCD (Coles et al., 2008). However, people with OCD often suffer from their symptoms and actively seek treatment as they consider these symptoms at odds with personal self-image (i.e., ego-dystonic). In contrast, subjects with OCPD consider symptoms as being consistent with their self-image (i.e., ego-syntonic) and rarely seek medical advice. Therefore, clarification of the relationship between OCD and OCPD would be highly advantageous if we are to understand these diseases better.

Decision-making is a cognitive skill that integrates environmental information to allow for beneficial outcomes. In a previous study, Knight divided decision-making into two broad categories: decision-making under risk and decision-making under ambiguity (Knight, 1921). Numerous reports have since documented the fact that OCD patients predominantly show impairments in decision-making under ambiguous situations but not in risky situations, and that this deficit is considered a major causative factor for patient distress (Starcke et al., 2010; Kim et al., 2015; Zhang et al., 2015a, b). The Iowa Gambling Task (IGT) is a widely used paradigm for measuring decision-making under ambiguity. Decision processing in IGT involves multiple mental processing, in which emotional cognitive processing plays an important role. During the task, the decision-making process leads to implicit emotional signal coding in subjects and guides the choice preference for cards (Vries et al., 2008). Previous studies that have used the IGT task for OCD subjects have demonstrated that decision-making under ambiguity is indeed deficient, thus leading to a more frequent choice of disadvantageous options. Further investigation of the physiological indicators found that OCD patients exhibit weaker skin conductance responses compared to healthy people. The reason for this phenomenon is that they are limited by their stronger emotions (Paolo et al., 2012). Moreover, this deficit is also found in relatives, indicating an underlying genetic propensity (Cavedini et al., 2010; Da et al., 2011). In contrast, few studies have examined decision-making in OCPD subjects. No significant difference was detected between OCPD subjects and healthy controls when using the Cambridge Gamble Task (Fineberg et al., 2015; Grant and Chamberlain, 2019). Alternatively, OCPD subjects exhibit a longer delay in decision-making compared to OCD and control subjects; this is consistent with the excessive self-control behavior observed in OCPD subjects (Pinto et al., 2014). Only a few publications have investigated decision-making in OCPD subjects and compared this with OCD subjects. Consequently, we know little about this process in these two sets of subjects, particularly with regards to decision-making under ambiguity.

Numerous neuroimaging studies have investigated the neural basis of decision-making under ambiguity. This has led to wide recognition of the pathophysiology model of OCD, involving the cortico-striato-thalamocortical (CSTC) circuits (Roth et al., 2007; van den Heuvel et al., 2015). Furthermore, neuroimaging studies have suggested that the prefrontal cortex area is involved in the pathophysiology of OCD (Nakao et al., 2014). In one study, Reetz et al. (2008) indicated that gray matter volume in multiple brain regions, such as the prefrontal cortex, cingulate, and the insula, of OCPD patients, was reduced compared to healthy controls. These lines of evidence suggested abnormalities in the prefrontal cortex of OCD and OCPD subjects. The neurological processes underlying decision-making under ambiguity can also be investigated by recording event-related potentials (ERPs) from the scalp, specifically the ERP component of feedback-related negativity (FRN). The FRN is an important indicator of an evaluative signal for outcome potency and outcome expectations (Gehring and Willoughby, 2002), and is important for successful decision-making (Zendehrouh, 2015). Simultaneous electroencephalogram (EEG) and functional magnetic resonance imaging (fMRI) recordings identified the anterior cingulate cortex (ACC) and medial prefrontal cortex as the major source of FRN (Gehring and Willoughby, 2002; Smith et al., 2015; Bluschke et al., 2018). Although many studies of OCD and OCPD have been driven by the hypothesis that psychopathology arises from persistently enhanced error signals, inconsistencies still exist in both experimental methods and results. Most studies reported larger FRN amplitudes in OCD patients, and increased FRN amplitude was considered as a biomarker or endophenotype for OCD (Endrass and Ullsperger, 2014; Klawohn et al., 2014; Riesel et al., 2014). One study found that subclinical obsessive-compulsive groups showed larger FRN amplitudes, along with impaired external monitoring capabilities (Zhu et al., 2014), while another study, using a learning task, found that OCPD showed smaller FRN amplitudes compared to controls (O’Toole et al., 2012). Although many studies focus on a neural mechanism under decision-making, the neurophysiological similarities or differences between OCD and OCPD in decision-making under ambiguity are still unclear.

Therefore, the present study aimed to compare behavior performance and the neural mechanisms involved with decision-making under ambiguity between OCD and OCPD subjects using a modified IGT task and ERPs methods. Based on the excessive control of uncertain scenarios and excessive emotional problems in subjects with obsessive-compulsive symptoms, we first hypothesized that both OCD and OCPD subjects would show impaired decision-making under ambiguity. Second, we observed the FRN component during the IGT task. The FRN component was chosen because it is more closely related to responses of error feedback from the external environment, which was also manipulated in our study. Therefore, we hypothesized that FRN amplitudes would be larger in OCD and OCPD subjects compared to controls due to over-monitoring in these subjects.

## Materials and Methods

### Participants

The OCD group consisted of 24 young patients (age = 19–22 years; 19.71 ± 0.33) recruited from the Mental Health Center of Anhui Province in Hefei, China. OCD was confirmed by at least two psychiatrists according to DSM-5. The Yale-Brown Obsessive-Compulsive Scale (Y-BOCS) was administered to all patients to evaluate the severity of symptoms, and those with a total severity score ≥ 16 were diagnosed with OCD. Thirteen of these patients did not use any drugs. Ten patients were being treated with selective serotonin reuptake inhibitors (SSRIs), two of which received SSRIs combined with antipsychotics. The remaining case was only taking antipsychotics.

Nineteen young people (age = 18–23 years old; 19.95 ± 0.21) who met our specific research criteria were included in the OCPD group. At first, a total of 654 undergraduate students of Anhui Medical University completed the OCPD subscale of the Personality Diagnostic Questionnaire-4+ (PDQ-4+), a self-reporting questionnaire in which each item is based on the eight listed DSM-IV-TR criteria for OCPD. The Chinese version of the PDQ-4+ achieves satisfying levels of reliability and validity (*r*_xx_ = 0.50–0.80, *r*_SB_ = 0.50–0.93, Cronbach’s = 0.56–0.78; Yang et al., 2002). Second, 26 subjects who scored more than four points were invited to take the Structured Clinical Interview for DSM-4 Axis II Personality Disorders. Eventually, 19 individuals met OCPD criteria, excluding those combined with OCD; these judgments were made by two psychiatrists.

Twenty-six normal control (NC) subjects (age = 18–25 years; 20.35 ± 0.30) were chosen from the pool of students with a PDQ-4+ score ≤ 3 and matched with OCD and OCPD cases by gender, handedness, age, and years of education. We excluded candidates undergoing pharmacotherapy (i.e., those taking a mind-changing medication), those with other psychiatric disorders according to the DSM-5, and those with a family history of psychotic disorders.

Also, we used the Hamilton Anxiety Rating Scale (HAMA-14 scores ≤ 14) and the Hamilton Depression Rating Scale (HAMD-17 scores ≤ 17) to investigate all subjects. We also used the Padua Inventory-Washington State University Revision (PI-WSUR) Chinese Version adapted by our research team which achieves satisfying levels of reliability and validity (Cronbach’s = 0.90; Pang et al., 2009). The PI-WSUR included 39 items assigned to five content categories: obsessional thoughts about harm to self/others, obsessional impulses to harm self/others, contamination obsessions and washing compulsions, checking compulsions, and dressing/grooming compulsions. Subjects presenting with a history of traumatic head injury, substance abuse, or neurological diseases, were also excluded. The background tests and characteristics of each group are summarized in Table 1.

**Table 1:** Background tests and characteristics in the three groups.

	OCD group (*n* = 24)	OCPD group (*n* = 19)	NC group (*n* = 26)	*χ*^2^/*F* value	*p* value
Sex	16/8	7/12	17/9	4.813	0.090
Handedness	22/1/1	19/0/0	26/0/0	3.862	0.425
Age	19.71 ± 0.33	19.95 ± 0.21	20.35 ± 0.30	1.281	0.284
Education	12.88 ± 0.40	13.63 ± 0.16	13.46 ± 0.14	2.140	0.126
HAMA	8.67 ± 0.72^ab^	2.89 ± 0.60	1.35 ± 0.24	32.362	<0.001**
HAMD	9.62 ± 1.00^ab^	3.26 ± 0.63	1.27 ± 0.26	25.538	<0.001**
Y-BOCS	20.50 ± 1.49	–	–	–	–
PI-WSUR	36.96 ± 3.57^b^	39.42 ± 4.00^c^	16.27 ± 2.24	16.195	<0.001**

Data are shown as mean ± SE. Annotations: OCD, obsessive-compulsive disorder; OCPD, Obsessive-compulsive personality disorder; NC, normal control; HAMA, Hamilton Anxiety Rating Scale; HAMD, Hamilton Depression Rating Scale; Y-BOCS, Yale-Brown Obsessive Compulsive Scale; PI-WSUR, Padua Inventory-Washington State University Revision; **p < 0.01. ^a^Significant difference between the OCD group and the OCPD group. ^b^Significant difference between the OCD group and NC group. ^c^Significant difference between the OCPD group and the NC group.

Written informed consent was obtained from all participants. All study procedures were approved by the Anhui Medical University Ethics Committee and conducted according to the Helsinki Declaration (1975 and subsequent revisions). The approval reference number was 2017012.

### Materials and Procedures

**Figure 1:**
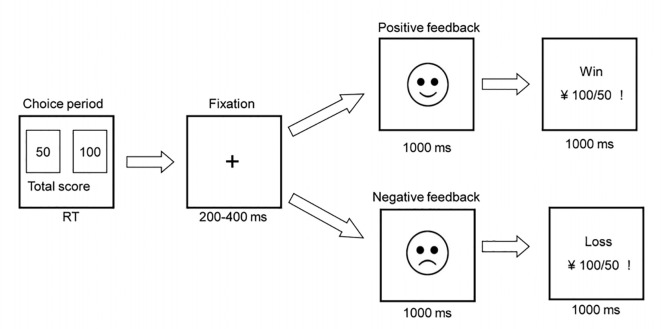
The sequence of performance in a single trial using the revised Iowa Gambling Task (IGT).

All subjects completed the modified IGT task, as described in a previous study (Zhu et al., 2014). Two alternative stimuli (decks) were presented on a computer screen at the beginning of each trial with a monetary value in RMB: a 50-yuan bet (left deck) and a 100-yuan bet (right deck). The winning ratios for 100-yuan and 50-yuan bets were 40 and 60%, respectively. Consequently, the 100-yuan bet was a disadvantageous option and the 50-yuan bet was an advantageous option. The sequence of the task was random. A blank screen with a central fixation cross appeared for 200−400 ms after selecting a bet. Then, an emoticon appeared for approximately 1,000 ms to represent whether the sequence was a loss (sad face) or a win (smiley face). Finally, the numerical information relating to the result of the bet appeared for 1,000 ms (Figure 1). Next, the decks appeared again to begin the next trial. The inter-trial interval was 1,200–1,500 ms. The task consisted of 300 trials and was divided into six blocks to investigate changes in decision-making success/failure. The net score for each block was obtained by subtracting the number of disadvantageous choices from the number of advantageous choices. Participants were told to win as much as possible with a seed amount of 1,000 yuan. Therefore, negative feedback occurred after losing money in the task. Each task took approximately 30 min to complete.

### Event-Related Potential Recording

EEG data were collected by a 64-channel Geodesic Sensor Net System (NeuroScan, Sterling, VA, USA) according to the international 10/20 system in an electro-acoustic shielded room. The forehead electrode was grounded and the left mastoid was used to connect an online reference electrode for all EEG channels. A horizontal electrooculogram (H-EOG) was obtained from the bilateral orbital rim. Vertical EOG was obtained from a supraorbital and infraorbital electrode on the left eye. Electrode impedances were maintained below 10 k. The EEG and EOG activities were amplified with a 0.01–100 Hz band-pass filter and the continuous sampling rate was 500 Hz/channel. The acquired signals were stored for subsequent off-line data analyses.

A self-coded MATLAB procedure, using functions from the EEGLAB environment, was adopted to process and analyze the EEG data offline (Delorme and Makeig, 2004). EEG data were re-referenced to the average of the EEG data arising from the left and right mastoid during the offline analysis process. The collected data were down-sampled to 250 Hz and then high-pass filtered at 1 Hz (FIR filter conducted using pop-eeg newfilt with the default parameters, using a cutoff frequency of 0.5 Hz, and 26 dB) to remove baseline shifts for independent component analysis (ICA; Delorme et al., 2012). Artifactual channels and non-brain electrodes were rejected by the clean raw data plug-in of EEGLAB, leaving an average of 58.35 [95%, (50, 60)] clean channels per participant. Continuous data were filtered and segmented from 1,000 ms before the feedback stimulus and increasing to 2,000 ms thereafter. Artifactual epochs were identified and removed on the basis of several criteria: (a) abnormal spectral characteristics of high frequency noise (rejspec; 20–40; <−35 or >35 dB); (b) abnormal trends (rejtrend; slope > 200 μV with *R*^2^ > 0.3); (c) abnormal amplitude (threshold −500 μV or + 500 μV); (d) improbable data using joint probability [jointprob, eight standard deviations (SD) for single-channel and 4 SDs for all channels]; and (e) abnormal distributions (rejkurt; eight SDs for a single channel and four SDs for all channels). Data from electrodes responsible for more than 10% of rejected epochs were eliminated from further analysis. Whole epochs were baseline-corrected to improve the reliability of the independent components (Groppe et al., 2009). Subsequently, epoched data were decomposed into maximally independent components using an extended infomax algorithm implemented by the runica function with default parameters in EEGLAB. Artifact components of HEOG, VEOG, and electromyogram (MEG) were identified and removed by the EEG_SASICA plug-in of EEGLAB combined with visual inspection (Chaumon et al., 2015). On average, there were 51.73 [95%, (45%, 58%)] components left per participant. On average, 3.2% of epochs were rejected in the NC group [95%, (1%, 9%)], 3.07% in the OCPD group [95%, (0%, 9%)], and 2.85% in the OCD group [95%, (0%, 9%)]. Rejection rates did not differ significantly among groups (*F*_(2,82)_ = 0.25, *p* = 0.78). The 250–350 ms time window in FRN was determined and five electrode points (Fz, FCz, Cz, CPz, and Pz) were selected for statistical analysis based on grand averages, topographical distribution, and previous studies (Hajcak et al., 2006; Cohen et al., 2007).

### Statistical Analysis

All statistical analyses were conducted using the SPSS software package (Version 16.0; SPSS Inc., Chicago, IL, USA). One-way analysis of variance (ANOVA) and the Chi-squared test were used to compare neuropsychological background and demographic data among groups. Repeated-measures ANOVA (RT-ANOVA) was used to analyze behavioral performance and ERP amplitude data in terms of the block (1–6), task condition (advantageous and disadvantageous), feedback (loss and win), electrodeposition (Fz, FCz, Cz, CPz, Pz), as within-subject factors, and group (OCD, OCPD, and NC) as between-subject factors. The degrees of freedom for the F ratios were adjusted in all analyses according to the Greenhouse-Geisser epsilon correction. Additionally, Pearson’s correlation was used to analyze the associations between PI-WSUR scores and task-related measures, including nets scores for the IGT and average amplitudes. All values are presented as the mean and standard error. The significance threshold was set at *p* < 0.05 (two-tailed).

## Results

### Group Differences in Demographics and Task Performance

Demographic variables are presented in Table 1. The three groups did not differ significantly from each other in terms of gender, handedness, age, and years of education. The OCD group had significantly larger scores in the HAMA and HAMD than the OCPD and NC groups (*p* < 0.001). Furthermore, the scores for the PI-WSUR in the OCD and OCPD groups were significantly different from those in the NC group (*p* < 0.001).

**Figure 2:**
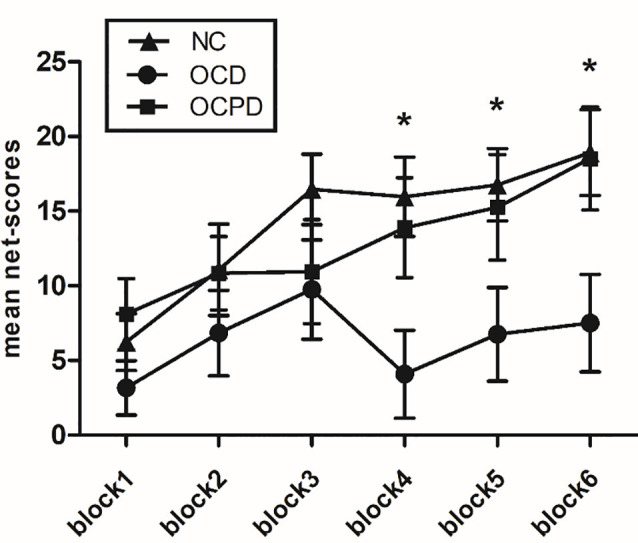
Net-scores for the six blocks during the IGT according to the three groups. *Significant difference between the OCD group and the NC group.

RT-ANOVA revealed significant main effects for group (*F*_(2,66)_ = 3.455, *p* < 0.05) and trial block (*F*_(5,330)_ = 8.810, *p* < 0.001) in terms of IGT net-scores. The OCD group performed worse than the NC (*p* = 0.015) and OCPD groups (*p* = 0.059), while there was no significant difference in performance between the OCPD and NC groups. The net-scores for each block gradually increased although there were significant differences among groups in the fourth (*p* = 0.011), fifth (*p* = 0.039), and sixth blocks (*p* = 0.018) blocks (Figure 2). Furthermore, there was a marginally significant interaction for both block and group (*F*_(10,330)_ = 1.733, *p* = 0.072). Net-scores differed significantly across blocks in the NC group (*F*_(5,155)_ = 3.319, *p* < 0.05), in which net-scores were significantly higher in blocks 3–6 (*p* < 0.01) compared to block 1, and the net-score of block 6 was significantly higher than that of block 2 (*p* = 0.033). Thus, the decision-making process improved significantly over time in the NC group. Conversely, the lack of change across blocks in the OCD group indicated a failure to improve decision-making through feedback. Notably, the OCPD group demonstrated a significantly higher net score in block 6 compared to block 1 (*p* = 0.021), thus indicating some preservation of learning from feedback.

### Group Difference in ERP Data

**Figure 3:**
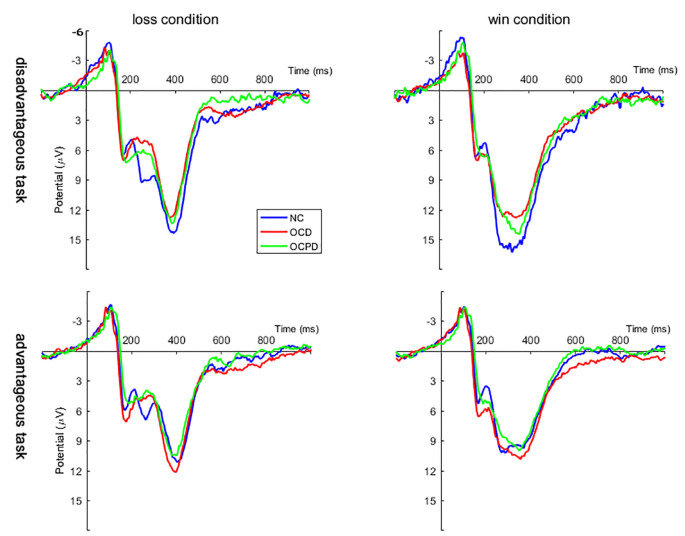
Grand averages evoked by loss and win feedback in disadvantageous and advantageous tasks at Fz, FCz, and Cz positions in the three groups.

Four-way mixed RT-ANOVA analysis revealed significant main effects for electrodeposition (*F*_(4,264)_ = 33.084, *p* < 0.001), task (*F*_(1,66)_ = 93.197, *p* < 0.001), and feedback (*F*_(1,66)_ = 80.212, *p* < 0.001) on FRN amplitude. The amplitude, which reached a maximum at Fz (9.524 ± 0.734 μV), was larger in the advantage condition compared to the disadvantage condition. Furthermore, the FRN amplitude was larger for the loss condition (10.216 ± 0.640 μV) than the win condition (13.880 ± 0.716 μV; Figure 3). There was also a significant interaction effect between feedback and group (*F*_(2,66)_ = 3.617, *p* < 0.05). The simple analysis revealed a significant difference between loss and win conditions in all three groups (*p* < 0.001); the largest difference was noted in the OCD group, followed by the OCPD group; the smallest difference was in the NC group.

**Figure 4:**
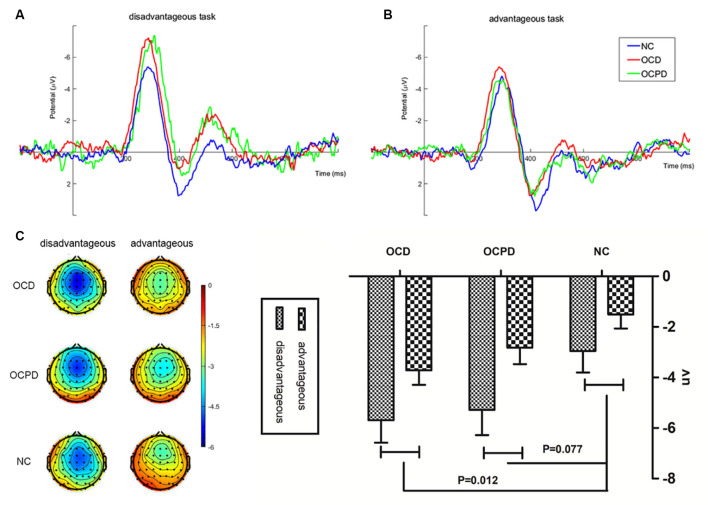
Feedback-related negativity (FRN) “loss-win” difference waves at FCz **(A)**, along with corresponding scalp topographies **(B)** and amplitude histograms **(C)** in disadvantageous and advantageous tasks in the three groups.

To better explain the feedback and group interaction effect, a three-way mixed RT-ANOVA was conducted on FRN amplitudes (dFRN) for the loss-win difference waveform. This analysis revealed significant main effects for group (*F*_(2,66)_ = 3.617, *p* < 0.05) and task (*F*_(1,66)_ = 31.145, *p* < 0.001). The OCD and OCPD groups exhibited significantly larger FRN amplitudes compared to the NC group (*p* = 0.012, *p* = 0.077; Figure 4). However, the difference between the OCD and OCPD groups was not significant. The differences in FRN waves, which were largest in the frontal central area (FCz, −4.377 ± 0.453 μV), were significantly larger when choosing the 100 yuan bet (−4.644 ± 0.528 μV) than the 50 yuan bet (−2.683 ± 0.343 μV).

### Relationship Between Clinical Characteristics and Task-Related Measures

Pearson’s correlation was used to investigate the relationships between clinical measures, task performance, and ERP data. The PI-WSUR scores for OC severity were significantly correlated with IGT net scores (*r* = −0.309, *p* = 0.010). Considering that depression and anxiety scores are also relevant to both OCD and OCPD, we conducted partial correlation analysis, using HAMA and HAMD scores as co-variables to remove the effects of anxiety and depression on the outcome. When accounting for these scores, the correlation between PI-WSUR and the IGT net scores was no longer significant (*r* = −0.194, *p* > 0.05). In addition, the IGT net scores were significantly and negatively correlated with HAMA (*r* = −0.251, *p* < 0.05) and HAMD (*r* = −0.316, *p* < 0.01). However, a significant negative correlation was observed between IGT net score and average FRN difference wave amplitude (*r* = −0.314, *p* < 0.05). Further analysis revealed that the significant correlation occurred only for the disadvantageous option (*r* = −0.363, *p* < 0.05).

## Discussion

This study aimed to investigate the processing of decision-making under ambiguous conditions between OCD and OCPD subjects using ERP methods. OCD patients showed a significantly impaired performance in the IGT task compared to the OCPD and NC groups. OCPD subjects showed medium performance when compared with the OCD and NC groups, thus indicating some preservation of an ability to learn from feedback in the OCPD group. Also, compared to the NC group, both OCD and OCPD groups demonstrated larger amplitudes for the loss-win FRN difference wave. These findings revealed that OCD and OCPD may share similar neural mechanisms for the feedback process although the behavior performance was worse in the OCD group compared to OCPD subjects.

Our analyses found that OCD patients showed impaired decision-making ability under ambiguous conditions. Our findings are consistent with other studies in that we report impaired decision-making under ambiguous conditions in OCD subjects (Cavedini et al., 2010; Grassi et al., 2015; Martoni et al., 2015). The IGT task used in this research was based on a long exploratory learning process. Compared with other tasks, the IGT task emphasizes the feedback learning process in decision-making (Bianchin and Angrilli, 2011). Decision-making under ambiguous conditions is dependent on feedback learning. The prerequisite for successful learning from feedback is performance monitoring (Yoshida and Seymour, 2014). The disadvantageous options were selected by NC subjects at the beginning of the task (blocks 1−3). With experience, these subjects would gradually realize that greater gain was accompanied by greater losses and thus learn to choose the advantageous option. This pattern of behavior is called loss avoidance or loss aversion (Kahneman and Tversky, 2000). In this task, OCD subjects preferred to choose higher and more immediate rewards, and completely ignored the long-term negative effects. OCD participants demonstrated serious impairments in decision-making that were not improved by experience. Compared with NC subjects, loss avoidance was not detected in the OCD subjects; this implies that OCD subjects did not change their behavior based on loss aversion. This phenomenon reflected obstacles in implicit feedback learning and over-monitoring in OCD subjects (Lei et al., 2015). Interestingly, we observed a phenomenon of loss avoidance in OCPD subjects during the IGT task. As we hypothesized, the final IGT performance did not differ between the NC and OCPD groups, but the improvement was slower in the OCPD group. A core feature of OCPD is non-adaptive perfectionism; these subjects focus on perfection and are often too rigid. They also tend to avoid negative emotions (Wheaton and Pinto, 2017). OCPD subjects are so sensitive to the negative emotions of loss that they would avoid greater losses. This behavior increases learning efficiency from feedback signals relative to the OCD subjects. A previous study demonstrated excessive self-control in OCPD subjects but not in OCD subjects with regards to delaying a decision-making task (Pinto et al., 2014). This may provide a basis for better adjustment capability in OCPD subjects compared to OCD subjects. In the present study, only OCD subjects showed decision-making obstacles. In contrast, the decision-making process in OCPD subjects was not impaired.

As expected, our electrophysiological findings showed that the amplitude of the FRN difference wave was higher in the OCD and OCPD groups. FRN is thought to reflect a reinforcement learning signal that is regulated by reward prediction which can quickly determine whether the result is better or worse than expected. An increase in FRN amplitude indicates a general increase in feedback-related brain activity (Hajcak et al., 2005). In the current study, the increased amplitude of FRN induced by performance in IGT was associated with heightened impulsivity. This association suggests that OCD and OCPD subjects cannot learn normally from negative feedback; this reflected the absence of developing a preference for the advantageous decks. Moreover, FRN reveals quantitative reward prediction errors in the monitoring of external feedback (Ullsperger et al., 2014). Compared with the NC group, the OCD and OCPD groups consistently showed an increased amplitude for the FRN difference wave, thus highlighting potential problems in the feedback monitoring mechanism. A larger FRN was also observed previously in OCD subjects during a time estimation task (Holroyd et al., 2006) and in a flanker task (Hanna et al., 2018). Another study reported a larger FRN amplitude in subclinical obsessive-compulsive subjects when taking the revised IGT (Zhu et al., 2014). Increased FRN amplitudes represent a hyperactive error signal in the brain that is often manifested by obsessive-compulsive symptoms such as perfectionism, uncertainty, and doubt, thus triggering repetitive or habitual actions. However, there are also some inconsistencies. A previous study used a probabilistic reinforcement learning task in which correct feedback was given with specified probability after the correct response; the authors of this study reported that subjects with OCD showed no differences in terms of FRN (Nieuwenhuis et al., 2005). Another research study observed similar outcomes in terms of obsessive-compulsive symptomatology in NCs (Gründler et al., 2009). Another study used a learning task in which appropriate feedback was given for every correct answer; the authors of this study reported that OCD and OCPD subjects exhibited a significantly reduced FRN when compared to NCs (Endrass et al., 2013). The reasons for these different outcomes may be related to the type of task and internal/external controllability (Endrass and Ullsperger, 2014). However, the task used in the present study was different than those used in these previous studies in that it was internally controllable. This means that the subjects underestimated the error rate of the task, thus resulting in a large difference between expectation and outcome. This, in turn, led to a larger FRN. Collectively, our present findings provide evidence for a deficient feedback monitoring process in both OCD and OCPD subjects.

Surprisingly, although OCD and OCPD subjects did not exhibit similar behavioral performances, we found similarities in their electrophysiological responses. From the perspective of the ERPs result, the amplitude of the FRN waves increased in both the OCD and OCPD groups when compared with the NC group. As mentioned above, this result was consistent with our hypothesis. Both OCD and OCPD might result in over-monitoring during the decision-making process, thus indicating certain similarities between these two subjects. However, from the perspective of behavioral performance, only the OCD group showed obstacles when deciding ambiguous conditions. According to the reinforcement learning theory, FRN represents an evaluating signal and appears when the results are worse than expected during decision making; the FRN is thought to precede behavioral adjustments (Endrass et al., 2013). Based on this interpretation, the completion of the IGT task relied on implicit FRN electrophysiological activities and behavioral adjustment processes. Only after the implicit processes were completed, would external behavior be demonstrated? Aside from these expectations, we can also interpret our results from an emotional perspective. During the feedback process, the emotion was clearly an important factor that could not be ignored. Indeed, some previous studies have shown impaired IGT performance in patients with major depressive disorder, and in non-clinical subjects with a more intense negative mood (Must et al., 2006; Suhr and Tsanadis, 2007). We found that the levels of anxiety and depression in the OCD group were significantly higher in OCPD subjects, as revealed by data acquired from the HAMA and HAMD scales. When considered emotion as a factor, the relationship between PI-WSUR scores and IGT net scores disappeared. Furthermore, IGT net scores were significantly and negatively correlated with HAMA and HAMD scales. Based on this, we speculated that if people with OC symptoms experienced more negative emotions, then decision-making behavior performance would be worse than for healthy people. This speculation was mainly based on the fact that OCPD patients believed that their symptoms were consistent with their self-image, so they might experience far fewer negative emotions than the OCD group. This had a smaller impact on behavioral performance; consequently, they performed in a manner that was more similar to healthy people when decision-making under ambiguous situations. This novel finding might provide a theoretical basis for the clinical treatment of obsessive-compulsive symptoms.

There are several limitations to our work that should be considered. First, OCPD should be divided into two dimensions (order/control and hoarding/indecision); the execution performance of these two dimensions may differ (Riddle et al., 2016). Second, the OCD subjects selected for this study were taking different medications and some had comorbid OCPD. It is widely known that medications can affect the preferences of OCD subjects during the IGT task (Long et al., 2015). The co-existence of OCD and OCPD results in more severe impairments in terms of executive function when compared to OCD without OCPD (Pinto et al., 2015). Research should be continued in more patients who have only OCD or OCPD and are not taking medication. Third, our research only involved young OCD and OCPD subjects. Several studies have found that OCD patients with comorbid OCPD have experienced a longer illness compared with OCD patients without OCPD, thus suggesting that age may have potential effects on decision-making ability (Diaferia et al., 1997; Coles et al., 2008; Garyfallos et al., 2010). Future studies should involve different age groups and large numbers of subjects for both OCD and OCPD. Fourth, in our study, we only included the FRN component in the analysis. However, other ERPs components that are not related to feedback were not reported, such as P300, an ERPs component that reflects the early attention distribution of feedback processing. Future studies are now needed to further explore more ERPs components that are related to feedback and emotional processing. Finally, the electrophysiological method used in this study had a low spatial resolution. Thus, a high spatial resolution brain imaging method, such as fMRI, should be implemented in future studies to better understand the neural mechanisms underlying decision-making in OCD and OCPD patients.

In conclusion, the present study demonstrated that OCD and OCPD subjects might share a similar neural mechanism among young people when decision-making under ambiguous circumstances, although OCPD showed better behavior performance compared to individuals with OCD patients. These results lend support to previous findings relating to obstacles in the feedback process in OCD subjects. Also, our study expanded our understanding of the similarities and differences in neural mechanisms between OCD and OCPD subjects.

## Data Availability Statement

The raw data supporting the conclusions of this article will be made available by the authors, without undue reservation.

## Ethics Statement

The studies involving human participants were reviewed and approved by the Anhui Medical University Ethics Committee and conducted according to the Helsinki Declaration (1975 and subsequent revisions). The approval reference number was 2017012. The patients/participants provided their written informed consent to participate in this study.

## Author Contributions

YL and LC: designed the research and interpreted the data and wrote the manuscript. YL: supervised data collection and analyzed the data. All authors contributed to the article and approved the submitted version.

## Conflict of Interest

The authors declare that the research was conducted in the absence of any commercial or financial relationships that could be construed as a potential conflict of interest.
